# Threshold values of brachial cuff-measured arterial stiffness indices determined by comparisons with the brachial–ankle pulse wave velocity: a cross-sectional study in the Chinese population

**DOI:** 10.3389/fcvm.2023.1131962

**Published:** 2023-07-13

**Authors:** Xujie Zhang, Yumin Jiang, Fuyou Liang, Jianping Lu

**Affiliations:** ^1^ Department of Engineering Mechanics, School of Naval Architecture, Ocean & Civil Engineering, Shanghai Jiao Tong University, Shanghai, China; ^2^ Physical Examination Center, Shanghai Sixth People’s Hospital, Shanghai Jiao Tong University School of Medicine, Shanghai, China; ^3^ World-Class Research Center “Digital biodesign and personalized healthcare”, Sechenov First Moscow State Medical University, Moscow, Russia

**Keywords:** arterial stiffness index, brachial cuff, arterial velocity-pulse index, arterial pressure-volume index, threshold value, brachial-ankle pulse wave velocity

## Abstract

**Background:**

Arterial Velocity-pulse Index (AVI) and Arterial Pressure-volume Index (API), measured by a brachial cuff, have been demonstrated to be indicative of arterial stiffness and correlated with the risk of cardiovascular events. However, the threshold values of AVI and API for screening increased arterial stiffness in the general population are yet to be established.

**Methods:**

The study involved 860 subjects who underwent general physical examinations (M/F = 422/438, age 53.4 ± 12.7 years) and were considered to represent the general population in China. In addition to the measurements of AVI, API and brachial-ankle pulse wave velocity (baPWV), demographic information, arterial blood pressures, and data from blood and urine tests were collected. The threshold values of AVI and API were determined by receiver operating characteristic (ROC) analyses and covariate-adjusted ROC (AROC) analyses against baPWV, whose threshold for diagnosing high arterial stiffness was set at 18 m/s. Additional statistical analyses were performed to examine the correlations among AVI, API and baPWV and their correlations with other bio-indices.

**Results:**

The area under the curve (AUC) values in ROC analysis for the diagnosis with AVI/API were 0.745/0.819, 0.788/0.837, and 0.772/0.825 (95% CI) in males, females, and all subjects, respectively. Setting the threshold values of AVI and API to 21 and 27 resulted in optimal diagnosis performance in the total cohort, whereas the threshold values should be increased to 24 and 29, respectively, in order to improve the accuracy of diagnosis in the female group. The AROC analyses revealed that the threshold values of AVI and API increased markedly with age and pulse pressure (PP), respectively.

**Conclusions:**

With appropriate threshold values, AVI and API can be used to perform preliminary screening for individuals with increased arterial stiffness in the general population. On the other hand, the results of the AROC analyses imply that using threshold values adjusted for confounding factors may facilitate the refinement of diagnosis. Given the fact that the study is a cross-sectional one carried out in a single center, future multi-center or follow-up studies are required to further confirm the findings or examine the value of the threshold values for predicting cardiovascular events.

## 1. Introduction

Arterial stiffness is a measure of the mechanical properties of the arterial wall, which determines the systolic and pulse pressures, thereby affecting the dynamic tension of arteries and the systolic load of the left ventricle (1). The stiffness of large elastic arteries increases progressively during aging as a consequence of wall thickening, changes in wall composition, and elastin degeneration (2). Many risk factors such as diabetes mellitus, hypertension, obesity, smoking, and kidney disease can accelerate arterial stiffening (3, 4). The value of arterial stiffness in predicting the risk of cardiovascular events has been extensively proven in both clinical and community-based cohorts (5–8). Moreover, the benefits of using arterial stiffness indices to guide the treatment of hypertension have also been demonstrated (9).

Over the past few decades, various non-invasive techniques for measuring arterial stiffness have been proposed. Among them, pulse wave velocity (PWV) measurement is widely accepted as the “standard” method due to its clear biophysical meaning (10–12). Two commonly used global PWV metrics are carotid-femoral PWV (cfPWV) and brachial-ankle PWV (baPWV) (10, 13). In eastern Asia, baPWV has been more widely used than cfPWV, enabling the accumulation of a large amount of data on the clinical implications of baPWV. Moreover, there are other arterial stiffness indices, such as carotid intima-media thickness (Carotid IMT), augmentation index (AIx), and cardio-ankle vascular index (CAVI), that have been proved to have potential clinical value (14–19). However, their applications in routine clinical practice remain challenging due to technical or economic reasons. Table 1 summarizes the strengths and limitations of various existing arterial stiffness indices. In this context, economically feasible and easier-to-use devices are desired. Such devices may facilitate routine screening for increased arterial stiffness in larger populations, thereby guiding the implementation of preventive interventions for individuals at an increased risk of cardiovascular disease (35, 36).

**Table 1:** Strengths and limitations of various arterial stiffness indices.

Indices	Strengths	Limitations
baPWV	• baPWV indicates the average stiffness of central and peripheral arteries in the upper and lower limbs (13) and is well-validated, providing good reproducibility (20). • baPWV has been demonstrated to be independently correlated with adverse cardiovascular events (20, 21) or the prognosis of chronic diseases like metabolic syndrome, type 2 diabetes, and kidney dysfunction (22–24).	• Some patients may feel uncomfortable due to long measurement time and postural requirement. • The equipment is pricey and necessitates skilled handling by professionals.
cfPWV	• cfPWV is considered the gold standard for arterial stiffness (10) and mainly reflects the stiffness of central arteries (13). • cfPWV has been shown to be a strong predictor of cardiovascular events and all-cause mortality (25) and has been extensively validated in various populations, including the elderly, diabetic patients, and patients with chronic kidney disease (26–28).	• Some patients might feel uneasy or embarrassed about exposing the inguinal area during the measurement of femoral pressure waveforms (13). • The measurement of cfPWV is technically challenging, and requires expertise and complex equipment.
Carotid IMT	• Carotid IMT is an intermediate phenotype for early atherosclerosis and can act as a surrogate marker for atherosclerosis (14). • Carotid IMT is associated with various cardiovascular risk factors and prevalent cardiovascular disease (CVD) (29), and is an independent predictor of cardiovascular events such as stroke and myocardial infarction (15).	• Carotid IMT assessment is a time-consuming and operator-dependent procedure (30), requiring training, experience, and standardized techniques. • Carotid IMT assessment can only be used to evaluate the carotid arteries, and cannot provide information on other areas of the vascular system.
AIx	• AIx is a measure of systemic arterial stiffness that is derived from the ascending aortic pressure waveform (16). • AIx can predict clinical events independently of peripheral pressures and has been shown to hold significant predictive value for cardiovascular events and all-cause mortality in various populations (17, 31).	• There is no standardized methodology for measuring AIx. • Arterial tonometry devices required to measure AIx are expensive and not widely available.
CAVI	• CAVI measures the stiffness of the arterial tree from the aorta to the ankle (32) and is independent of blood pressure changes during the measurements (33). • CAVI has been widely applied to assess high-risk populations, such as those at risk of developing or who have already developed coronary artery disease, cerebrovascular disease, diabetes mellitus, and chronic kidney disease (18, 19).	• CAVI cannot be accurately measured in patients with aortic stenosis, peripheral artery disease, or atrial fibrillation (34). • CAVI measurements should only be performed by trained medical professionals while the patient is in the supine position.

PASESA AVE-2000 (DAIWA Healthcare, Shenzhen, China) offers a fully automatic and rapid measurement of arterial stiffness, which can be easily implemented by clinicians or even by patients themselves. The compact and economical design of the device, which uses a single oscillatory brachial cuff to noninvasively measure physiological signals, makes it ideal for routine clinical use. The device provides two arterial stiffness indices, Arterial Velocity-pulse Index (AVI) and Arterial Pressure-volume Index (API), which respectively reflect the stiffness of the central arteries and brachial artery. The potential clinical value of AVI and API has been confirmed by many clinical studies. For example, AVI and/or API have been found to be significantly correlated with major adverse cardiac events during follow-up of outpatients (37), associated with the Framingham cardiovascular disease risk score in both outpatients and the general population (38, 39), and predicative of early coronary artery atherosclerosis in patients with suspected coronary disease (40) and cardiovascular risk in hypertensive patients (41). These studies demonstrated the clinical implications of AVI and API, but did not provide threshold values for identifying subjects with abnormally high arterial stiffness in the general population. Therefore, the main purpose of this study was to fill this gap. For this purpose, we collected data from adult participants who underwent a general health check at the physical examination center. The study cohort was comprised of individuals aged in a large range, including both healthy subjects and patients with cardiovascular diseases, and was therefore considered to be representative of the general population in China. The threshold values of AVI and API for diagnosing high arterial stiffness were determined by using baPWV as the reference. The results demonstrated that given proper threshold values, AVI and API could be utilized to identify individuals with increased arterial stiffness. On the other hand, the threshold values were gender-dependent and considerably affected by certain confounding factors.

## 2. Methods

### 2.1. Study population

A total of 860 subjects, who were aged between 20 and 91 years and visited the physical examination center of Shanghai Sixth People's Hospital for a general health check, were recruited. The study was approved by the ethics committee of the hospital. Each participant provided written informed consent after receiving detailed information about the study's goals and procedures. Before data collection, each participant completed a written questionnaire about their medical history, including hypertension, major cardiovascular diseases, type 2 diabetes mellitus, any other cardiovascular-related abnormalities, and medications. Notably, participants without any reported diseases on the questionnaire were categorized into a disease group if their health check results indicated the presence of cardiovascular-related diseases. In this study, healthy subjects were those who did not suffer from hypertension, type 2 diabetes mellitus, dyslipidemia, obesity, or other cardiovascular diseases based on the questionnaire and health check report.

### 2.2. Measurements of AVI, API and baPWV

The measurements of AVI, API and baPWV were conducted in a separate room with a controlled environmental temperature of approximate 25°C. Each participant was asked to rest for at least 10 min before the measurements were taken. Firstly, AVI and API were measured together with brachial systolic pressure, diastolic pressure, and heart rate, in a sitting position using a commercial medical device (PASESA AVE-2000, DAIWA Healthcare, Shenzhen, China). Subsequently, the participant was asked to rest in a supine position for at least 5 min, and then baPWV was measured using BP-203RPEIII (Omron, Dalian, China). The reliability and validity of the baPWV device have been confirmed by a previous study (20). In this study, a baPWV of >18 m/s, as recommended in the guidelines for non-invasive vascular function tests (42), was adopted as the threshold for diagnosing high arterial stiffness. Hereafter, we briefly introduce the measuring principles of AVI and API.

**Figure 1:**
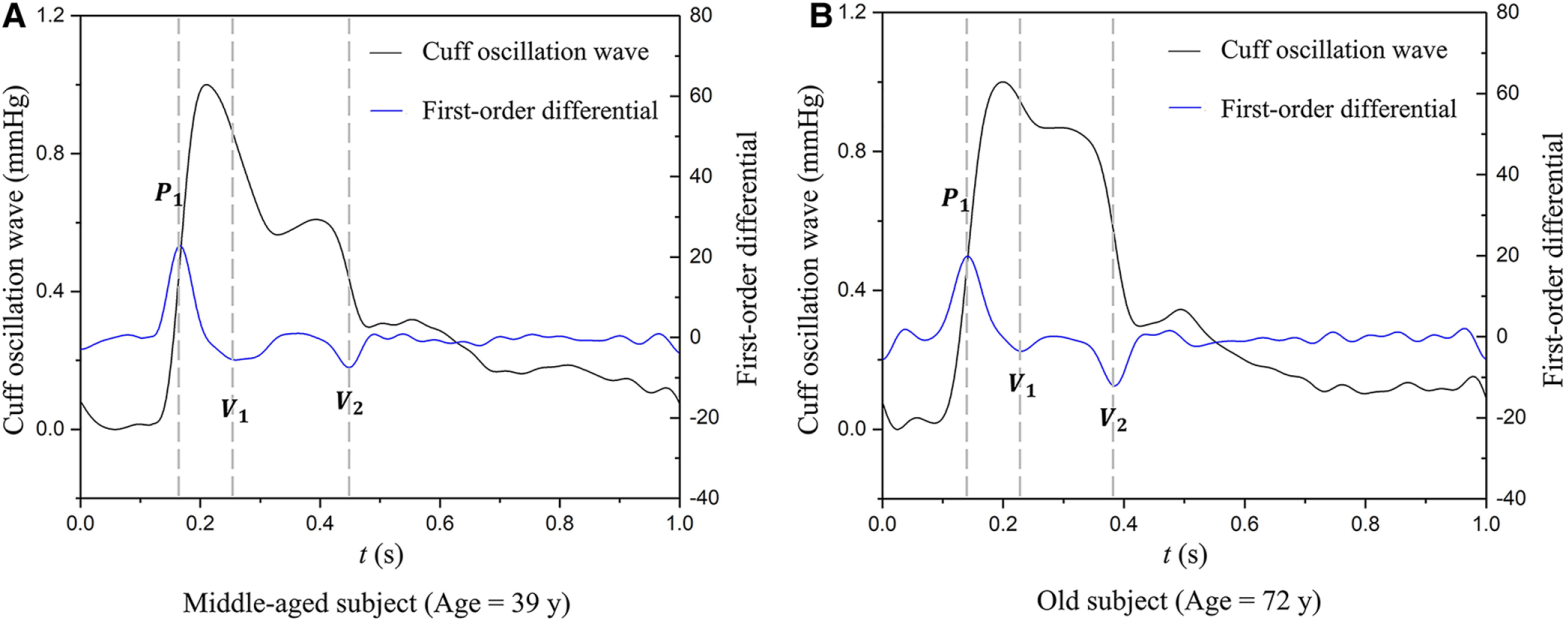
Examples of cuff oscillation waves (measured when the cuff operating pressure is higher than the brachial systolic pressure) and their first-order differentials used to calculate AVI: (**A**) data of a middle-aged subject, (**B**) data of an old subject. *P*_1_, the first peak; *V*_1_, the first valley; *V*_2_, the second valley.

AVI is calculated by analyzing the oscillation waves measured by a brachial cuff operating under high-pressure conditions (higher than the brachial systolic pressure). Figure 1 illustrates the cuff oscillation waves and their first-order differentials measured in a middle-aged subject and an old subject, respectively. It can be observed that the second valley of the differentiated cuff oscillation wave in the old subject is lower than that in the middle-aged subject, whereas the peak is comparable between the two subjects. AVI is defined as the ratio of the magnitude of the second valley (|*V*_2_|) to that of the peak (|*P*|) multiplied by a constant (*A*).

(1) $$\mathrm{AVI}=\;A\times |V_{2}|/|P|$$

**Figure 2:**
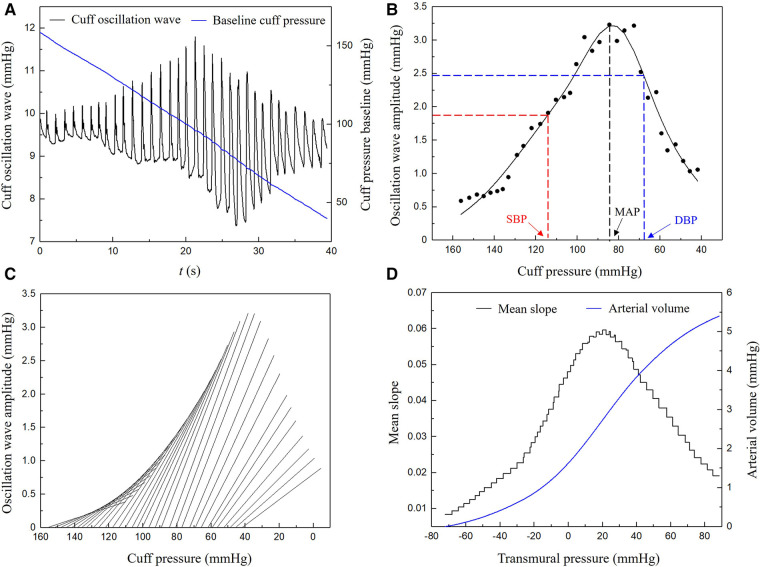
Procedure of deriving a pressure-vascular volume characteristic curve from measured time series cuff pressure: (**A**) baseline cuff pressure and cuff oscillation wave, (**B**) envelope curve and the estimation of brachial blood pressures, (**C**) local slopes of the cuff pressure-arterial volume characteristic curve, (**D**) arterial transmural pressure-volume characteristic curve. SBP, systolic blood pressure; DBP, diastolic blood pressure; MAP, mean blood pressure.

**Figure 3:**
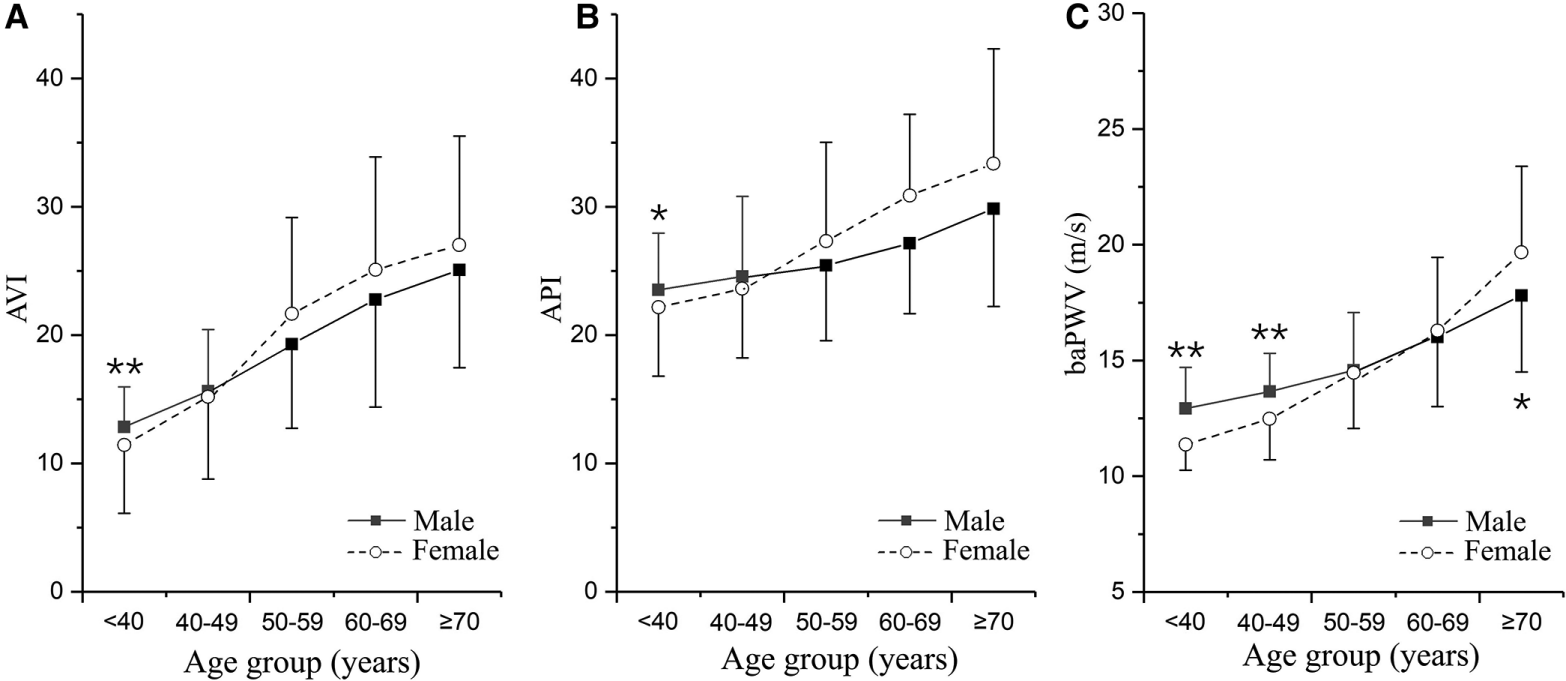
Changes in arterial stiffness indices with age differentiated by sex: (**A**) AVI, (**B**) API, and (**C**) baPWV. The plotted data are the means and standard deviations of arterial stiffness indices in different age groups. The numbers of males and females in the five age groups are as follows: <40 years, *n* = 56/49; 40–49 years, *n* = 116/116; 50–59 years, *n* = 113/130; 60–69 years, *n* = 96/100; and ≥70 years, *n* = 41/43. *p < 0.05, **p < 0.01 vs. male group.

API is calculated by analyzing the time series cuff oscillation waves monitored during the decrease of cuff pressure from a supra-systolic pressure level to a value lower than the diastolic pressure (43). The measuring principle is based on the biomechanical mechanism that the shape of the transmural pressure-area (or volume) curve of an artery is mainly determined by the stiffness of arterial wall when the transmural pressure is varied over a wide range. To construct this curve using noninvasively measured cuff signals, the following procedure was implemented: (1) digitally filter the original cuff pressure data to extract the time series baseline cuff pressure and oscillation component (i.e., oscillation waves) (Figure 2A); (2) construct the envelope curve based on the amplitudes of oscillation waves and estimate brachial blood pressures (Figure 2B); (3) calculate the local slopes of the cuff pressure—arterial volume characteristic curve (Figure 2C); and (4) reconstruct the arterial transmural pressure—volume characteristic curve by numerically integrating the slopes obtained in step 3) (Figure 2D). Finally, an arctangent function was used to fit the curve.

(2) f(x)=aarctan⁡(bx+c)+d

where the reciprocal of *b* was taken as an indirect measure of the vascular wall stiffness under low transmural pressure conditions. API is defined as

(3) API=X×1/b

where *X* is a constant used to scale up the magnitude of API.

For a more detailed introduction of the measuring principles of AVI and API, please refer to the Supplementary Material.

### 2.3. Other measurements

All participants had their height and weight measured, and their body mass indices (BMIs) were calculated to evaluate obesity. Blood samples were collected for laboratory examinations of indices considered to be related to diabetes mellitus or cardiovascular diseases, including fasting plasma glucose (FPG), glycated hemoglobin (HbA1c), total cholesterol (TC), triglycerides (TG), high-density lipoprotein cholesterol (HDL-C) and low-density lipoprotein cholesterol (LDL-C). The lipoprotein ratios (TC/HDL-C, LDL-C/HDL-C) were also calculated. In addition, urine samples were analyzed for microalbumin (UMAlb).

### 2.4. Statistical analysis

Baseline characteristics were presented separately for the male, female and pooled groups in form of either median and interquartile range or percentage. Statistical analyses were implemented in SPSS 24. Comparison of percentage values was performed using the Chi-square test. When comparing the quantitative data between the male and female groups, the student's *t*-test was employed if the data conform to a normal distribution, otherwise, the Mann–Whitney *U*-test was performed. Pearson or Spearman rank correlation analysis was applied to evaluate the correlations among AVI, API, baPWV and their correlations with other variables based on the results of normality test. The stepwise multiple linear regression analyses were carried out to evaluate the independent associations of AVI, API, baPWV with other bio-indices. Moreover, receiver-operating characteristic (ROC) curve analyses and covariate-adjusted ROC (AROC) analyses (44) were carried out to seek for the threshold values of AVI and API, where the sum of sensitivity and specificity was used as the criterion for evaluating diagnosis performance. Statistical significance was defined as *p* < 0.05.

## 3. Results

### 3.1. Characteristics of participants

Table 2 shows the diagnostic criteria for diseases and the disease-specific distributions of participants. The proportions of hypertension and type 2 diabetes in the study cohort were comparable to the prevalence of these diseases in Chinese adults as reported in the “2019 Report on Cardiovascular Health and Diseases in China and a national cross-sectional study on diabetes” (45) (hypertension: 30.8% vs. 33.5%; type 2 diabetes: 11.5% vs. 12.8%). Hence, the subjects involved in our study are roughly representative of the general Chinese population. The 860 participants constituted a pooled group, which was further divided into the male and female groups. The characteristics of participants in the three groups are presented in Table 3. There were no significant differences in demographic data between the male and female groups, except for BMI, DBP, MAP and HR. Serum lipid indices differed significantly between the male and female groups, with females having higher TC and HDL-C, and lower TG and lipoprotein ratios (TC/HDL-C, LDL-C/HDL-C). Overall, the proportion of females with dyslipidemia was much smaller than that of males. In addition, males had a significantly higher prevalence of hypertension and type 2 diabetes mellitus compared to females. If the subjects with hypertension were divided into the treated and untreated subgroups, SBP [139.96 ± 19.20 vs. 146.78 ± 17.69 (mmHg), *p* = 0.002], MAP [100.04 ± 16.49 vs. 104.41 ± 12.89 (mmHg), *p* = 0.031] and the three arterial stiffness indices [baPWV: 16.63 ± 3.23 vs. 17.74 ± 3.69 (m/s), *p* = 0.005; AVI: 21.81 ± 7.95 vs. 26.46 ± 8.64, *p* < 0.0001; API: 29.79 ± 8.06 vs. 32.69 ± 7.44, *p* < 0.0001] were all slightly lower in the treated subgroup, indicating the role of antihypertensive treatment in reducing arterial stiffness.

**Table 2:** Distributions of diseases in the studied population.

Diseases	*n* (%)	Diagnostic criteria
Hypertension	265 (30.8%)	Systolic blood pressure (SBP) > 140 mmHg, diastolic blood pressure (DBP) > 90 mmHg, or history of drug treatment for hypertension
Type 2 diabetes mellitus	99 (11.5%)	Fasting plasma glucose (FPG) ≥ 7.0 mmol/L, oral glucose tolerance test: two-hour plasma glucose ≥11.1 mmol/L, glycated haemoglobin (HbA1c) ≥ 6.5%, or history of treatment with insulin or an oral hypoglycemic agent
Major cardiovascular diseases	25 (2.9%)	History of diagnosed or treated cardiovascular diseases including but not limited to stroke, coronary heart disease, heart failure, or peripheral arterial disease

**Table 3:** Characteristics of the studied subjects.

	Male (*n* = 422)	Female (*n* = 438)	Pooled (*n* = 860)	*p* Value
Demographic data				
Age (years)	53.00 (45.00–62.00)	53.00 (46.00–62.00)	53.00 (45.00–62.00)	0.461
Gender (m/f %)	100.00/0.00	0.00/100.00	49.07/50.93	0.440
BMI (kg/m²)	24.39 (22.56–26.61)	22.99 (21.08–25.15)	23.66 (21.77–25.97)	<0.001***
SBP (mmHg)	127.00 (115.00–138.00)	124.00 (110.00–138.00)	125.00 (113.00–138.00)	0.079
DBP (mmHg)	78.00 (71.00–85.00)	73.00 (65.00–82.00)	76.00 (68.00–84.00)	<0.001***
MAP (mmHg)	94.33 (86.00–102.33)	90.00 (80.67–100.67)	92.67 (83.33–102.00)	<0.001***
HR (bpm)	78.00 (71.00–85.00)	72.00 (67.00–81.00)	72.00 (66.00–80.00)	0.029*
Laboratory data				
FPG (mmol/L)	5.11 (4.82–5.62)	5.11 (4.80–5.48)	5.11 (4.80–5.52)	0.195
HbA1c (%)	5.50 (5.40–5.80)	5.60 (5.40–5.80)	5.50 (5.40–5.80)	0.571
TC (mmol/L)	4.84 (4.23–5.44)	4.93 (4.36–5.60)	4.89 (4.31–5.52)	0.020*
TG (mmol/L)	1.74 (1.21–2.46)	1.31 (0.93–1.86)	1.49 (1.04–2.18)	<0.001***
LDL-C (mmol/L)	3.07 (2.58–3.67)	3.13 (2.56–3.71)	3.10 (2.57–3.68)	0.549
HDL-C (mmol/L)	1.15 (0.94–1.37)	1.41 (1.19–1.68)	1.28 (1.05–1.55)	<0.001***
TC/HDL-C	4.18 (3.45–4.97)	3.50 (2.91–4.28)	3.83 (3.10–4.65)	<0.001***
LDL-C/HDL-C	2.68 (2.06–3.29)	2.23 (1.71–2.84)	2.44 (1.86–3.11)	<0.001***
UMAlb (+/− %)	11.14/88.86	9.36/90.64	10.23/89.77	0.390
Clinical diagnosis				
Hypertension (treatment) (%)	34.83 (62.59)	26.94 (55.08)	30.81 (59.25)	0.012* (0.002**)
Type 2 diabetes mellitus (%)	14.69	8.45	11.51	0.004**
Cardiovascular disease (%)	3.79	2.97	3.37	0.504
Dyslipidemia (%)	52.61	34.02	43.14	<0.001***
Obesity (%)	12.09	8.90	10.47	0.128

BMI, body mass index; SBP, systolic blood pressure; DBP, diastolic blood pressure; MAP, mean blood pressure; HR, heart rate; FPG, fasting plasma glucose; HbA1c, glycated haemoglobin; TC, total cholesterol; TG, triglycerides; LDL-C, low-density lipoprotein cholesterol; HDL-C, high-density lipoprotein cholesterol; UMAlb, urine microalbumin. Data are presented in form of median (IQR) or percentage.

* *p* < 0.05.

** *p* < 0.01.

*** *p* < 0.001 vs. male group.

### 3.2. Differences of AVI, API and baPWV between male and female groups

The Mann–Whitney *U*-test was performed to compare AVI, API and baPWV between the male and female groups in different age brackets. The mean values and standard deviations (SDs) of the arterial stiffness indices grouped by sex and age brackets are presented in Figure 3. AVI, API and baPWV all increased with age. In the age bracket of <40 years, the mean values of AVI and API in the female group were significantly lower than those in the male group (AVI: *p* < 0.0001, API: *p* = 0.03), and the mean values of baPWV in the female group remained lower than those in the male group until the age increased over 50 years (<40 years: *p* < 0.0001, 40–49 years: *p* < 0.0001). In the age brackets of >50 years, all the arterial stiffness indices increased more rapidly with age in the female group, leading to obviously higher AVI, API and baPWV in older females.

### 3.3. Results of correlation analysis and regression analysis

**Figure 4:**
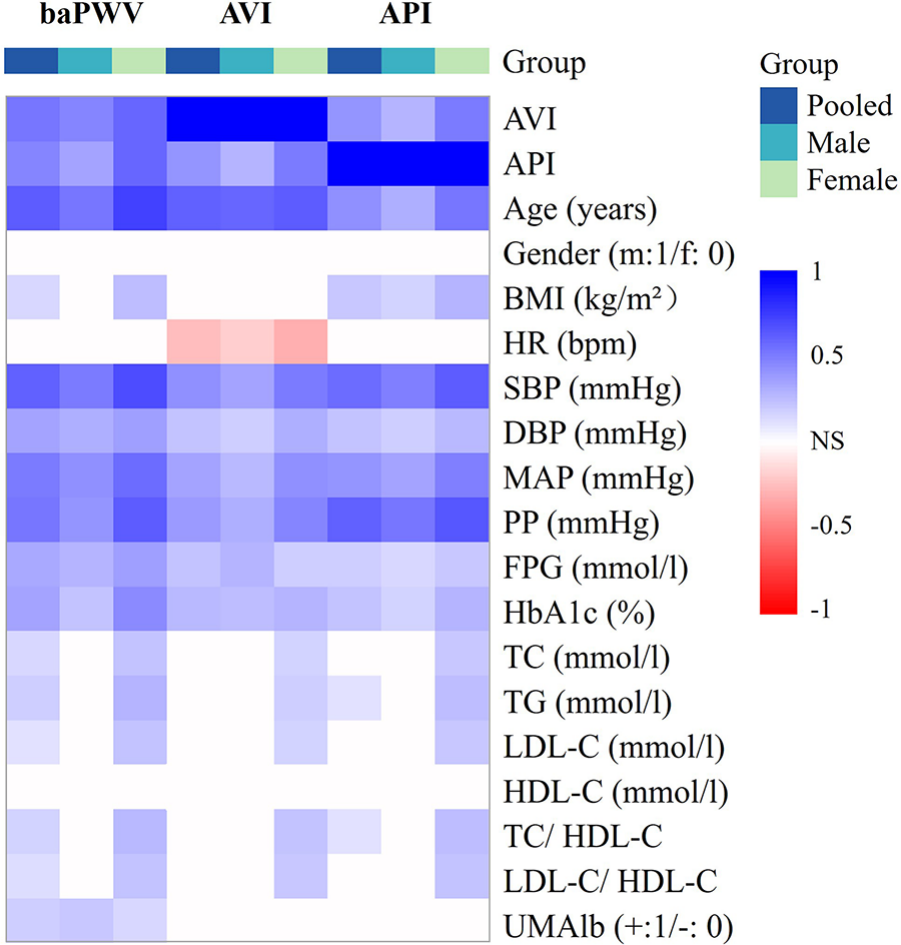
Heat map of the correlations among arterial stiffness indices and their correlations with other bio-indices.

In this study, since all datasets did not exhibit a strict normal distribution, the Spearman correlation analysis was performed in conjunction with Bonferroni correction for *p*-value thresholds to examine the pairwise correlations among AVI, API and baPWV, and their correlations with other bio-indices in the male, female and pooled groups, respectively. Figure 4 presents the results in the form of a heat map where gradually changing colors denote the strength of correlation while the white color indicates the lack of a statistically significant correlation between two indices. Herein, the statistical significance was defined as *p* < 0.00263 (0.05/*n*, where *n* denotes the number of selected variables in Bonferroni correction).

AVI and API were both positively correlated with baPWV, and the correlations were especially strong in the female group. AVI, API and baPWV were most strongly correlated with age, PP, and age & SBP, respectively (*r* > 0.6) in the pooled population, and the strong correlations were more evident in the female group. The stepwise multiple regression analysis was carried out to further identify the associated factors of AVI, API and baPWV for the male, female and pooled groups, respectively. The results showed that SBP, age, HR and FPG were positively associated with baPWV in all groups, gender and TG were positively associated with baPWV in the pooled group, whereas UMA1b was associated with baPWV only in the male group. Age, MAP and HR were the main relevant factors of AVI, and FPG was associated with AVI only in the male group. PP, BMI and age were significantly associated with API in the male group, whereas PP, age, SBP and HDL-C were significantly associated with API in the female group (Table 4).

**Table 4:** Results of stepwise multiple regression analysis for arterial stiffness indices with respect to bio-indices.

baPWV									
	Pooled			Male			Female		
Adjusted R-squared value	0.623			0.518			0.699		
Variables	*β*	SE	*p*	*β*	SE	*p*	*β*	SE	*p*
SBP (mmHg)	0.065	0.004	<0.001	0.049	0.006	<0.001	0.069	0.005	<0.001
Age (years)	0.116	0.007	<0.001	0.107	0.009	<0.001	0.121	0.010	<0.001
HR (bpm)	0.051	0.006	<0.001	0.056	0.009	<0.001	0.049	0.009	<0.001
FPG (mmol/L)	0.241	0.054	<0.001	0.196	0.072	0.006	0.339	0.083	<0.001
Gender (m:1/f: 0)	0.387	0.143	0.007	NS	NS	NS	NS	NS	NS
TG (mmol/L)	0.127	.056	0.024	NS	NS	NS	NS	NS	NS
UMA1b (+:1/−: 0)	NS	NS	NS	0.739	0.331	0.026	NS	NS	NS
**AVI**									
	Pooled			Male			Female		
Adjusted R-squared value	0.370			0.307			0.438		
Variables	*β*	SE	*p*	*β*	SE	*p*	*β*	SE	*p*
Age (years)	0.285	0.022	<0.001	0.245	0.027	<0.001	0.299	0.034	<0.001
MAP (mmHg)	0.178	0.020	<0.001	0.110	0.028	<0.001	0.213	0.028	<0.001
HR (bpm)	−0.141	0.021	<0.001	−0.105	0.028	<0.001	−0.206	0.031	<0.001
FPG (mmol/L)	NS	NS	NS	0.432	0.216	0.046	NS	NS	NS
**API**									
	Pooled			Male			Female		
Adjusted R-squared value	0.431			0.310			0.498		
Variables	*β*	SE	*p*	*β*	SE	*p*	*β*	SE	*p*
PP (mmHg)	0.177	0.027	<0.001	0.232	0.025	<0.001	0.142	0.037	<0.001
BMI (kg/m^2^)	0.382	0.068	<0.001	0.446	0.091	<0.001	NS	NS	NS
Age (years)	0.078	0.020	<0.001	0.081	0.025	0.001	0.117	0.031	<0.001
Gender (m:1/f: 0)	−1.675	0.426	<0.001	NS	NS	NS	NS	NS	NS
SBP (mmHg)	0.074	0.020	<0.001	NS	NS	NS	0.117	0.026	<0.001
HR (bpm)	−.043	0.018	0.017	NS	NS	NS	NS	NS	NS
HDL-C (mmol/L)	NS	NS	NS	NS	NS	NS	−2.666	0.816	0.001

*β*, regression coefficient; SE, standard error.

### 3.4. Results of ROC analysi**s** and AROC analysis

**Figure 5:**
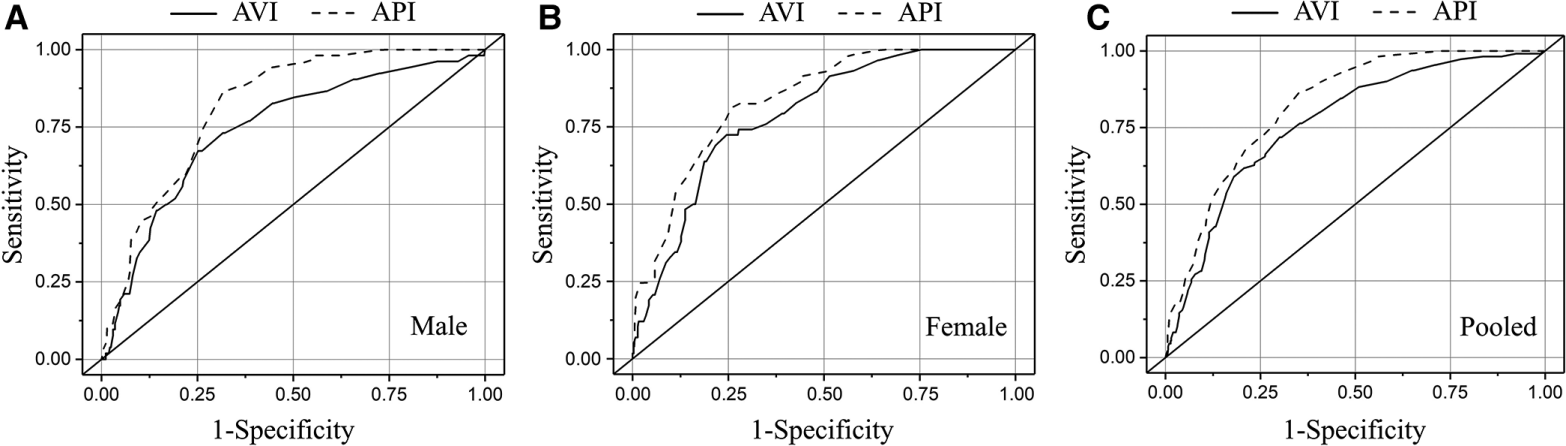
Results of ROC analyses on the diagnosis performances of AVI and API in different groups: (**A**) male, (**B**) female, and (**C**) pooled.

**Figure 6:**
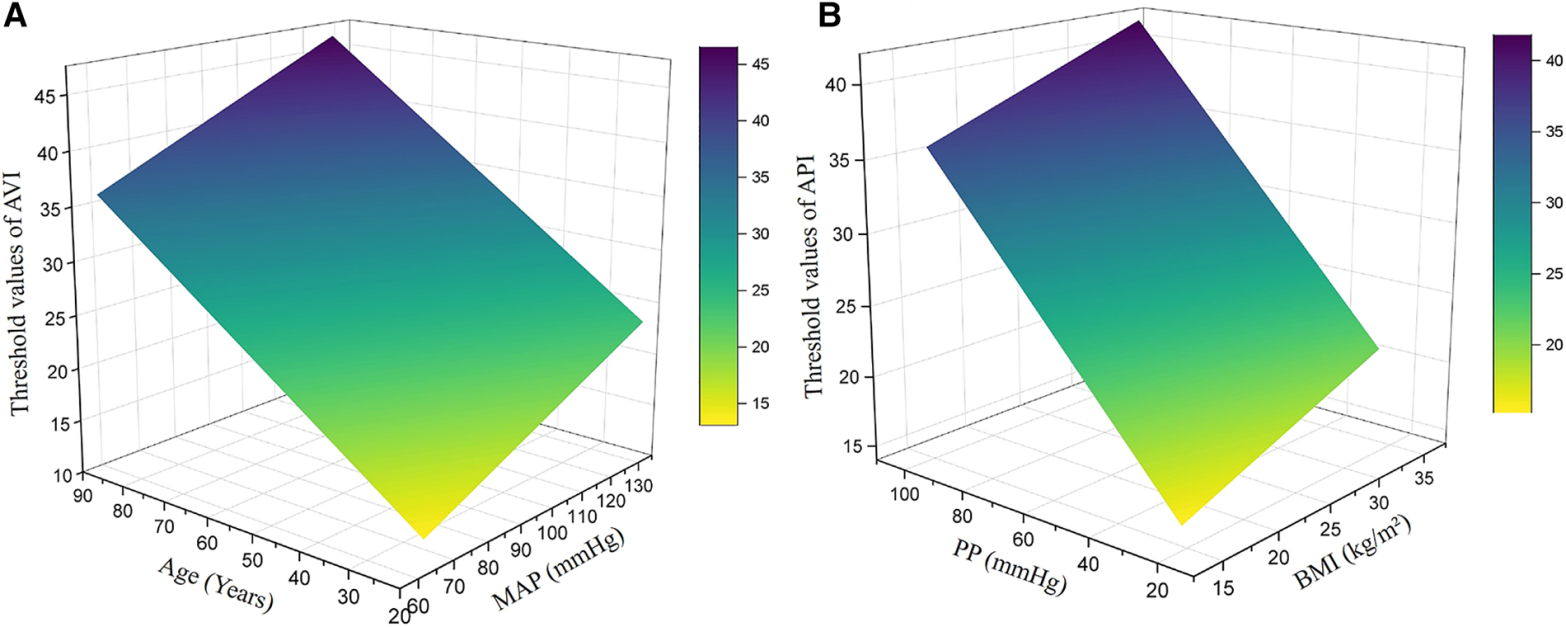
Threshold values of AVI and API in the pooled group after adjustment for major covariates: (**A**) AVI, (**B**) API. The major covariates were age and MAP for AVI, and PP and BMI for API.

In order to determine the optimal threshold values of AVI and API for diagnosing high arterial stiffness, we took the results of diagnosis with baPWV as the reference to perform ROC analysis. In consideration of potential sex differences, the threshold values of AVI and API were determined separately for the male, female and pooled groups. Figure 5 plots the results of ROC analyses with optimized threshold values of AVI and API, and the quantitative data are summarized in Table 5. The values of AUC (95% CI) for diagnosis with AVI/API were 0.745/0.819, 0.788/0.837, and 0.772/0.825 in males, females, and all subjects, respectively. In all groups, the larger AUC values of API indicate its better diagnosis performance than AVI, which can also be judged from the ROC curves plotted in Figure 5. In addition, the threshold values of AVI and API were both larger in females than in males, which led to mildly improved diagnosis performance for the female group. Furthermore, we carried out covariate-adjusted ROC (AROC) analysis (44) to investigate the influences of confounding factors (those identified by the multiple regression analysis for AVI or API). We set two confounding factors associated most closely with AVI or API as covariates and analyzed the data of the pooled group only. Figure 6 shows that the threshold values of AVI and API changed markedly with the variations in covariate values. Specifically, the threshold value of AVI increased markedly with age and moderately with MAP, whereas the threshold value of API increased evidently with PP and mildly with BMI.

**Table 5:** Threshold values of AVI and API and the corresponding results of ROC analysis on the diagnosis performance.

	AUC (95% CI)	SE	Threshold	Sensitivity	Specificity	PPV	NPV	Accuracy	*p*
Male									
AVI	0.745 (0.672, 0.817)	0.037	21	0.673	0.749	0.273	0.942	0.739	<0.0001
API	0.819 (0.747, 0.891)	0.037	27	0.865	0.683	0.278	0.973	0.705	<0.0001
Female									
AVI	0.788 (0.729, 0.857)	0.035	24	0.724	0.755	0.311	0.947	0.751	<0.0001
API	0.837 (0.766, 0.898)	0.031	29	0.807	0.750	0.326	0.963	0.757	<0.0001
Pooled									
AVI	0.767 (0.717, 0.817)	0.026	21	0.718	0.700	0.260	0.944	0.702	<0.0001
API	0.825 (0.779, 0.872)	0.024	27	0.862	0.649	0.264	0.970	0.676	<0.0001

SE, standard error; AUC, area under curve; PPV, positive predictive value; NPV, negative predictive value.

## 4. Discussion

Increased arterial stiffness, an important risk factor for cardiovascular diseases, remains under-diagnosed in the general population. Despite the existence of well-validated devices for measuring arterial stiffness indices such as baPWV or cfPWV, these devices are usually costly, require professional operations, and take over 10 min to complete measurement, which significantly hampers their wide use in routine medical practice. The present study attempted to determine the threshold values of arterial stiffness indices (i.e., AVI and API) measured by a cuff-based device (PASESA AVE-2000) in the general Chinese population by referring to the diagnosis results with baPWV. The threshold values of AVI and API giving rise to optimal diagnosis performance (evaluated by AUC values in ROC analysis) in the entire cohort were found to be 21 and 27, respectively, with corresponding AUC values of 0.767 and 0.825. If evaluated separately for the female and male groups, the diagnosis performances could be slightly improved in the female group if the threshold values of AVI and API were increased to 24 and 29, respectively. These results imply that AVI and API measured by the cuff-based device have the potential to be used as substitutes for baPWV in the assessment of arterial stiffness. In particular, since the device is portable and easy to use like a general blood pressure device, it may be employed to perform large-scale screening for individuals with high arterial stiffness in daily medical activities. Given the well-established value of arterial stiffness in predicting cardiovascular events, early diagnosis of high arterial stiffness would help initiate early preventative interventions (e.g., changes in lifestyle or medical treatment) that have been widely recognized as cost-effective strategies for reducing the risk of cardiovascular diseases (46, 47). AVI and API may also be used as complementary bio-indices for ambulatory PWV and ambulatory blood pressure (ABP), which can be measured outside the hospital and have been proved to have specific value in evaluating antihypertensive treatment or identifying individuals with an increased cardiovascular risk (48, 49), to strengthen the reliability of diagnosis.

Previous clinical studies have demonstrated the potential of threshold values of AVI and API for stratifying the cardiovascular risk of patients. For instance, setting the threshold value of AVI to 27 was found capable of predicting major adverse cardiac events (MACEs) in outpatients in Japan (37). In Japanese patients suffering from heart failure with preserved ejection fraction (HFpEF), AVI > 30 was predictive of high *E*/*e*′ ratio and high levels of high-sensitivity cardiac troponin T (hs-cTnT) (50). With respect to API, patients with API > 32 were found to suffer from a significantly increased risk of MACEs during a mean follow-up period of 769 ± 275 days (37). Similarly, a study on Chinese hypertensive patients with HFpEF (41) demonstrated that an increase in AVI over 27 or API over 31 was associated with an increased risk of total cardiovascular events during follow-up. In our study, the relatively low threshold values of AVI and API may be explained by the following reasons: (1) we used baPWV > 18 m/s as the criterion for judging high arterial stiffness, which is associated with but not equivalent to the risk of cardiovascular events, and (2) our study was focused on the general population whose heath conditions are overall better than those of the outpatients investigated by previous studies. Nevertheless, adopting relatively conservative threshold values for AVI and API may facilitate the identification of individuals with potential cardiovascular risk from the general population for whom early preventive interventions would be especially necessary and beneficial.

Our study demonstrated that AVI and API differed evidently between females and males, especially with respect to their changes with age and the threshold values. While the threshold values of AVI and API in the male group were the same with those determined for the entire cohort, they were 3 and 2 units higher respectively in the female group. Accordingly, the diagnosis performance in the female group was slightly improved if higher threshold values of AVI and API were used (see Table 5), which implies that adopting gender-specific threshold values for AVI and API may facilitate more accurate identification of females with abnormally high arterial stiffness. The results may be explained by the different sensitivities of AVI, API and baPWV to age between females and males. As shown in Figure 3, AVI and API increase more rapidly with age in females than in males, especially when ages are higher than 50 years. Such gender differences are also observed for baPWV, but are relatively small. Mechanisms underlying the observations are complex and may involve multiple factors. Hormones, especially estrogen, play an important role in modulating arterial stiffness. Estrogen has been found to stimulate the release of nitric oxide, thereby contributing to improving the elasticity of blood vessels and reducing arterial stiffness (51, 52). However, the protective role of estrogen is markedly attenuated in postmenopausal women (aged over 45–55 years) (53) due to decreased estrogen secretion, causing arterial stiffness to increase rapidly with age (54, 55). It has been found that isolated systolic hypertension (ISH) preferentially affects old women (56), which may also contribute to the increase of arterial stiffness via its roles in promoting vascular thickening and fibrosis (57). In addition, aged women are more susceptible to the decrease in muscle mass and accumulation of visceral fat, both of which have been proved to be associated with increased arterial stiffness in postmenopausal women (58, 59). These factors underline the need to take gender-related characteristics into account when diagnosing and managing arterial stiffness.

Many bio-indices were found to be correlated with AVI and API, which could be confounding factors compromising the validity of the threshold values of AVI and API determined by the general ROC analysis. To address the issue, we performed covariate-adjusted ROC (AROC) analyses on the pooled group, with major confounding factors identified by the multiple regression analysis being set as the covariates. The results revealed that age and PP were the two confounding factors that most evidently affect the threshold values of AVI and API, respectively. Specifically, the higher the age and PP were, the higher the adjusted threshold values of AVI and API became. The influence of age on the threshold value of AVI can be explained by the high sensitivity of AVI to age as indicated by the data presented in Figure 3A. As for PP, it significantly affects the threshold value of API because PP is a variable directly involved in the calculation of API (43). Therefore, adopting confounding factor-adjusted threshold values for AVI and API may be beneficial to the refinement of diagnosis. For instance, using higher threshold values for aged individuals with naturally high arterial stiffness and individuals with high brachial PP due to physiological rather than pathological factors can reduce the risk of misdiagnosis that might lead to unnecessary interventions or treatments. Despite the potential benefits, using threshold values adjusted for various confounding factors to perform diagnosis for large populations in routine medical practice could be challenging, as it significantly increases the complexity and time required for data collection and classification. In fact, current clinical guidelines or expert consensuses often recommend the use of a single threshold value for each physiological index, such as blood pressure or arterial stiffness.

The study has some potential limitations. Firstly, it was conducted in a single center and involved a relatively small population. In particular, the diagnostic accuracy of AVI and API using the threshold values was not validated in an independent sample, and hence the generalizability of our findings to other populations remains unclear. Secondly, the study, as a cross-sectional one, could not address the usefulness of the threshold values in predicting future cardiovascular events. To address the limitations, future multi-center or follow-up studies on larger populations would be required. Thirdly, in comparison with baPWV, cfPWV and carotid intima-media thickness (IMT) can provide a more specific assessment of central arterial stiffness, and hence may be better comparators of AVI. However, their measurements were not available in the physical examination center where we collected the data, and this limitation might be addressed in our future studies by introducing cfPWV devices and collaborating with clinicians in the ultrasound department. Fourthly, we fixed the threshold value of baPWV at 18 m/s for diagnosing high arterial stiffness. However, different threshold values of baPWV have been proposed in the literature (60–63). Therefore, it remains debatable whether choosing a different threshold value for baPWV might yield a more reasonable determinant for the threshold values of AVI and API.

## 5. Conclusions

The threshold values of AVI and API measured by a cuff-based device for diagnosing high arterial stiffness in the general Chinese population have been established through comparisons with baPWV. The performances of diagnosis (evaluated by AUC) were basically acceptable (AUC > 0.76 with AVI, and AUC > 0.82 with API) in the pooled group, which could be further improved if gender-specific threshold values were applied to the female group. In the meantime, the study revealed that age and PP had significant influences on the threshold values of AVI and API, respectively, indicating the potential of using confounding factor-adjusted threshold values to refine diagnosis. On the other hand, the findings of the study are limited by the relatively small sample and absence of follow-up data, which would be addressed by future multi-center or follow-up studies on larger populations.

## Data Availability

The original contributions presented in the study are included in the article/**Supplementary Material**, further inquiries can be directed to the corresponding authors.
