# Supplementary material for: Threshold values of brachial cuff-measured arterial stiffness indices determined by comparisons with the brachial–ankle pulse wave velocity: a cross-sectional study in the Chinese population
Source: Front Cardiovasc Med. 2023 Jul 13;10:1131962. doi: 10.3389/fcvm.2023.1131962 (PMC10381930; doi:10.3389/fcvm.2023.1131962)
Supplement: Supplementary file 1 [file Table1.pdf]

Supplementary Material

Threshold values of brachial cuff-measured arterial stiffness indices determined by comparisons with the brachial–ankle pulse wave velocity: A cross-sectional study in the Chinese Population

Xujie Zhang*

*** Correspondence:** Fuyou Liang: fuyouliang@sjtu.edu.cn. Jianping Lu: 18930177452@163.com.

1 Measuring Principles of AVI and API

1.1 AVI

AVI is calculated by analyzing oscillation waves measured by a brachial cuff operating under high pressure conditions (higher than the brachial systolic pressure), with specific algorithms. Previous theoretical and clinical studies have revealed that following the increase in central arterial stiffness during aging, there is a noticeable rise of the secondary peak or inflection point in the systolic portion of the cuff oscillation wave followed by a rapid drop in pressure, whereas the wave component in early systole is less affected (1-2). These features can be highlighted by computing the first-order differential of the cuff oscillation wave. Figure 1 illustrates the cuff oscillation waves and their first-order differentials measured in a middle-aged subject and old subject respectively. One can observe that each differentiated cuff oscillation wave exhibits one peak (in early systole) and two valleys (the first one in mid systole and the second one in late systole), and that the second valley in the old subject is lower than in the middle-aged subject, whereas the peak is comparable between the two subjects. AVI is then defined as the ratio of the magnitude of the second valley ($|V_2|$) to that of the peak ($|P|$) multiplied by a constant (A).

$$\mathrm{AVI} = \mathrm{A} \times |V_2|/|P| \tag{1}$$

A previous study has proved that the cuff oscillation wave measure by a brachial cuff operating at high pressure has a similar shape to the blood pressure wave in the brachial artery (1). Therefore, by referencing to the formation process of the brachial arterial blood pressure wave, we can explore the biomechanical mechanisms underlying the characteristics of cuff wave. Physiologically, an increase in the stiffness of the aorta and central arteries (e.g., during aging) will accelerate the speed of pulse wave propagation in the arterial system which causes most reflected pressure waves originating from peripheral vascular beds to arrive at the brachial artery in mid systole to elevate the second peak of the pressure wave. As a result, the intensity of reflected pressure waves arriving at the brachial artery in late systole when the blood pressure is in the descent phase will attenuate, which causes the blood pressure to decrease more rapidly, leading to the appearance of a deeper second valley (V_2) on the differentiated wave. In contrast, P, as the largest positive pressure slope in early systole when the blood pressure increases rapidly, is closely associated with the intensity of the accident pressure wave, which is determined mainly by the contractility of the left ventricle (3). Therefore, AVI will theoretically increase with the stiffening of the aorta and central arteries. It may also increase if the systolic function of the left ventricle severely impairs (i.e., the value of P decreases significantly) (4).

1.2 API

API is calculated by analyzing the time series cuff oscillation waves monitored during the decrease of cuff pressure from a supra-systolic pressure level to a value lower than the diastolic pressure (5). The key biomechanical mechanism of the measuring principle is that the shape of the transmural pressure-area (or volume) curve of an artery is closely related to the stiffness of arterial wall. Specifically, when the arterial wall is softer, the slope of the curve becomes steeper, particularly when the transmural pressure is low and the arterial wall is in an unloaded state (6). To construct a transmural pressure-volume curve for the brachial artery beneath the cuff, the following procedure was implemented: 1) digitally filter the original cuff pressure data to extract the time series baseline cuff pressure and oscillation component (i.e., oscillation waves) (Figure 2A); 2) construct the envelope curve based on the amplitudes of oscillation waves and estimate brachial blood pressures (Figure 2B); 3) calculate the local slopes of the cuff pressure - arterial volume characteristic curve (Figure 2C); and 4) reconstruct the arterial transmural pressure - volume characteristic curve by numerically integrating the slopes obtained in step 3) (Figure 2D). Finally, an arctangent function was used to fit the curve.

$$f(x) = a\arctan(bx + c) + d \quad (2)$$

where coefficients a, b, c, and d are determined through data fitting. The value of b is the main determinant of the curve slope in the low transmural pressure zone. For example, when b is small, the curve slope is low, corresponding to stiff vascular wall, whereas when b is large, the curve slope is high, corresponding to soft vascular wall. Thus, the reciprocal of b can be taken as an indirect measure of the vascular wall stiffness under low transmural pressure conditions. Therefore, API is defined as

$$\mathrm{API} = \mathrm{X} \times 1/b \quad (3)$$

where X is a constant used to scale up the magnitude of API.

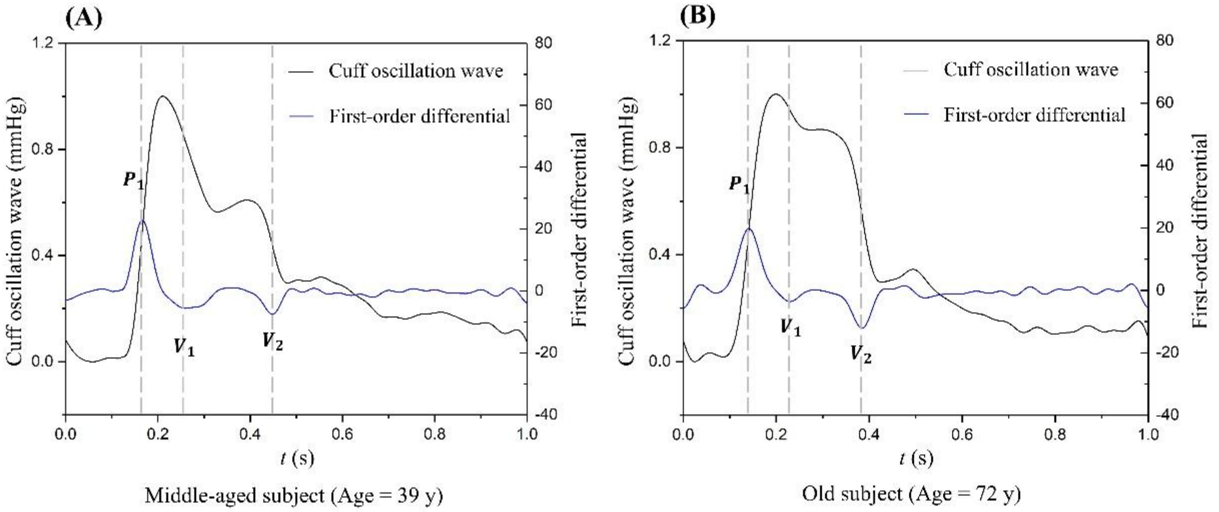

Figure 1 Examples of cuff oscillation waves (measured when the cuff operating pressure is higher than the brachial systolic pressure) and their first-order differentials used to calculate AVI: (A) data of a middle-aged subject, (B) data of an old subject. P_1: the first peak, V_1: the first valley, V_2: the second valley.

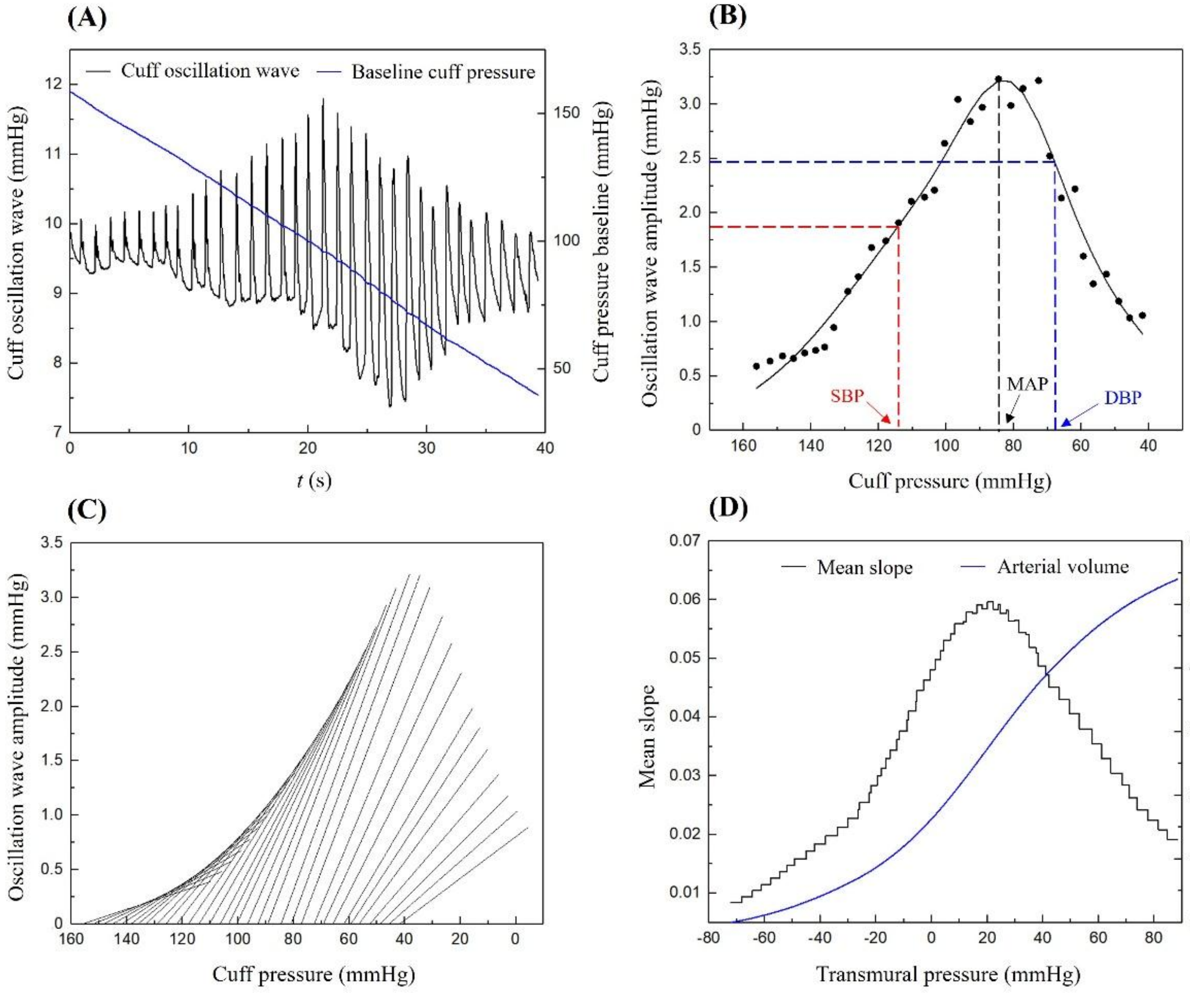

Figure 2 Procedure of deriving a pressure-vascular volume characteristic curve from measured time series cuff pressure: (A) baseline cuff pressure and cuff oscillation wave, (B) envelope curve and the estimation of brachial blood pressures, (C) local slopes of the cuff pressure-arterial volume characteristic curve, (D) arterial transmural pressure-volume characteristic curve. SBP, systolic blood pressure; DBP, diastolic blood pressure; MAP, mean blood pressure.

Reference

1. Liang F, Takagi S, Himeno R, Liu H. A Computational Model of the Cardiovascular System Coupled with an Upper-Arm Oscillometric Cuff and Its Application to Studying the Suprasystolic Cuff Oscillation Wave, Concerning Its Value in Assessing Arterial Stiffness. *Computer methods in biomechanics and biomedical engineering* (2013) 16(2):141-57.

2. Sueta D, Yamamoto E, Tanaka T, Hirata Y, Sakamoto K, Tsujita K, et al. The Accuracy of Central Blood Pressure Waveform by Novel Mathematical Transformation of Non-Invasive Measurement. *International journal of cardiology* (2015) 189:244-6.

3. Tartiere JM, Logeart D, Beauvais F, Chavelas C, Kesri L, Tabet JY, et al. Non - Invasive Radial Pulse Wave Assessment for the Evaluation of Left Ventricular Systolic Performance in Heart Failure. *European journal of heart failure* (2007) 9(5):477-83.

4. Zhang Y, Yin P, Xu Z, Xie Y, Wang C, Fan Y, et al. Non-Invasive Assessment of Early Atherosclerosis Based on New Arterial Stiffness Indices Measured with an Upper-Arm Oscillometric Device. *The Tohoku journal of experimental medicine* (2017) 241(4):263-70.

5. Komine H, Asai Y, Yokoi T, Yoshizawa M. Non-Invasive Assessment of Arterial Stiffness Using Oscillometric Blood Pressure Measurement. *Biomedical engineering online* (2012) 11(1):1-12.

6. LIANG F, LIU H, TAKAGI S. The Effects of Brachial Arterial Stiffening on the Accuracy of Oscillometric Blood Pressure Measurement: A Computational Model Study. *Journal of Biomechanical Science and Engineering* (2012) 7(1):15-30.
